# Whole-Exome Sequencing in the Differential Diagnosis of Primary Adrenal Insufficiency in Children

**DOI:** 10.3389/fendo.2015.00113

**Published:** 2015-08-05

**Authors:** Li F. Chan, Daniel C. Campbell, Tatiana V. Novoselova, Adrian J. L. Clark, Louise A. Metherell

**Affiliations:** ^1^Centre for Endocrinology, William Harvey Research Institute, Barts and The London School of Medicine and Dentistry, Queen Mary University of London, London, UK

**Keywords:** adrenal insufficiency, whole-exome sequencing, FGD, genetics

## Abstract

Adrenal insufficiency is a rare, but potentially fatal medical condition. In children, the cause is most commonly congenital and in recent years a growing number of causative gene mutations have been identified resulting in a myriad of syndromes that share adrenal insufficiency as one of the main characteristics. The evolution of adrenal insufficiency is dependent on the variant and the particular gene affected, meaning that rapid and accurate diagnosis is imperative for effective treatment of the patient. Common practice is for candidate genes to be sequenced individually, which is a time-consuming process and complicated by overlapping clinical phenotypes. However, with the availability, and increasing cost effectiveness of whole-exome sequencing, there is the potential for this to become a powerful diagnostic tool. Here, we report the results of whole-exome sequencing of 43 patients referred to us with a diagnosis of familial glucocorticoid deficiency (FGD) who were mutation negative for *MC2R*, *MRAP*, and *STAR* the most commonly mutated genes in FGD. WES provided a rapid genetic diagnosis in 17/43 sequenced patients, for the remaining 60% the gene defect may be within intronic/regulatory regions not covered by WES or may be in gene(s) representing novel etiologies. The diagnosis of isolated or familial glucocorticoid deficiency was only confirmed in 3 of the 17 patients, other genetic diagnoses were adrenal hypo- and hyperplasia, Triple A, and autoimmune polyendocrinopathy syndrome type I, emphasizing both the difficulty of phenotypically distinguishing between disorders of PAI and the utility of WES as a tool to achieve this.

## Introduction

The hypothalamic–pituitary–adrenal (HPA) axis is essential in the physiological response to stress and illness. Such external stimuli trigger the production of ACTH from corticotroph cells in the anterior pituitary, under the control of hypothalamic CRH and AVP, which acts on the ACTH receptor [also known as MC2R] on the surface of adrenal cells to elicit the production of glucocorticoids (1). The adrenal gland is composed of a medulla which secretes catecholamines and a cortex which comprises three layers: the zona glomerulosa which produces mineralocorticoids, the zona fasciculata which produces glucocorticoids (cortisol in humans), and the zona reticularis which produces adrenal androgens. Adrenal insufficiency is a serious medical condition that is invariably fatal unless diagnosed and treated early. Adrenal insufficiency is classified into primary, direct impairment of the adrenal gland to secrete cortisol, or secondary, impaired ACTH secretion from the pituitary gland. Primary adrenal insufficiency (PAI) can be acquired or congenital, the acquired group including infectious, traumatic, or drug-induced causes. Congenital PAI encompasses disorders such as congenital adrenal hyperplasia (CAH) (2–4), familial glucocorticoid deficiency (FGD) (5), and autoimmune polyglandular syndrome type I (APS-1) (6) as well as many others (listed in Table 1).

**Table 1:** **List of disorders and gene mutations identified with primary adrenal insufficiency (PAI) as a core characteristic**.

Gene name and symbol	Disorder	OMIM number	Extra adrenal manifestation	No of exons
21-Hydroxylase (*CYP21A2*)	CAH	201910	Ambiguous genitalia, hirsutism	10
11-B Hydroxylase (*CYP11B1*)	CAH	202010	Ambiguous genitalia, hypertension	9
17-a hydroxylase (*CYP17A1*)	CAH	202110	Ambiguous genitalia, hypertension, delayed puberty	8
Nuclear receptor subfamily 5A1 (*NR5A1*)	AI	184757	Gonadal dysgenesis, XY sex reversal	7
Nuclear receptor subfamily 0B 1 (*NR0B1*)	AHC	300200	Hypogonadotrophic hypogonadism in males (Duchenne muscular dystrophy, glycerol deficiency if part of Xp21 deletion)	2
P450 (cytochrome) oxidoreductase (*POR*)	CAH	201750	Antley-Bixler syndrome with genital anomalies	15
3-beta hydroxysteroid dehydrogenase (*HSD3B2*)	CAH	201810	Ambiguous genitalia	4
Cytochrome P450 11A1 (*CYP11A1*)	AI	613743	XY sex reversal	9
Steroidogenic acute regulatory protein (*STAR*)	LCAH/FGD	201710/609197	XY sex reversal	7
Alacrima achalasia adrenal (*AAAS*)	Triple A	231550	Alacrima, achalasia, deafness, cognitive impairment, hyperkeratosis	16
Autoimmune regulator (*AIRE*)	APECED/APS1	240300	Hypoparathyroidism, immune deficiency	14
Cyclin-dependent kinase inhibitor 1C (*CDKN1C*)	IMAGE	614732	IUGR, metaphyseal dysplasia, genital anomalies	3
ATP-binding cassette, sub-family D, member 1 (*ABCD1*)	X-ALD	300100	Muscle weakness, cognitive degeneration, blindness, spasticity, quadriparesis	11
Melanocortin 2 receptor (*MC2R*)	FGD	202200	N/A	1
MC2R accessory protein (*MRAP*)	FGD	607398	N/A	4
Mini chromosome maintenance deficient (*MCM4*)	FGD	609981	NK cell deficiency, short stature, chromosomal instability	22
Nicotinamide nucleotide transhydrogenase (*NNT*)	FGD	614736	N/A	22
Thioredoxin reductase 2 (*TXNRD2*)	FGD	-	N/A	7
Glutathione peroxidase 1 (*GPX1*)	FGD	-	N/A	2
Peroxiredoxin 3 (*PRDX3*)	FGD	-	N/A	7

*Key: OMIM, online Mendelian inheritance in man; CAH, congenital adrenal hyperplasia; AI, adrenal insufficiency; AHC, adrenal hypoplasia congenital; LCAH, lipoid congenital adrenal hyperplasia; FGD, familial glucocorticoid deficiency; APECED/APS1, autoimmune polyendocrinopathy-candidiasis-ectodermal dystrophy/autoimmune polyendocrinopathy syndrome type I; IMAGE, IUGR, metaphyseal dysplasia, genital anomalies; X-ALD, X-linked Adrenoleukodystrophy; IUGR, intrauterine growth retardation; NK, natural killer; N/A, not applicable*.

Children with PAI present with signs and symptoms resulting from low serum cortisol (such as failure to thrive, hypoglycemia, and lethargy) and from high plasma ACTH levels (hyperpigmentation – which is variable and can be dependent on ethnic origin/MC1R status). Biochemical analysis in cases of PAI will often reveal a low morning serum cortisol concentration (<80 nmol/l) paired with elevated plasma ACTH levels (>45 pmol/l), due to lack of negative feedback (7, 8). Such biochemical indicators change depending on the clinical scenario, for example, cortisol measurements maybe within normal range but inappropriately low for the given situation, for example during sepsis (9). Measurement of urea and electrolytes, plasma renin activity or concentration, and aldosterone concentration will determine the presence of mineralocorticoid deficiency. An ACTH stimulation test is often undertaken in cases of suspected adrenal insufficiency, where a synthetic form of ACTH, (ACTH [1–24], Synacthen), is administered and plasma cortisol measured 30 and 60 min later. PAI is excluded if serum cortisol concentrations are above 500 nmol/l following 250 μg of Synacthen administered IV or IM (10).

**Figure 1:**
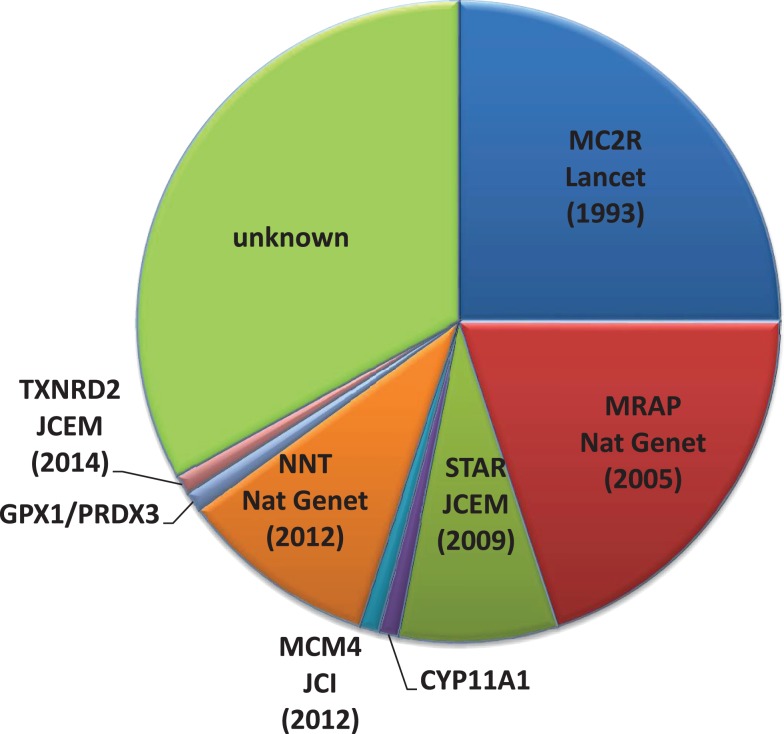
**Pie chart indicating the percentage of FGD cases due to gene mutations in the Melanocortin 2 receptor (*MC2R*), MC2R accessory protein (*MRAP*), Mini chromosome maintenance deficient 4 (*MCM4*), Nicotinamide nucleotide transhydrogenase (*NNT*), Steroidogenic acute regulatory protein (STAR), Cytochrome p450 11A1 (CYP11A1), Thioredoxin reductase 2 (*TXNRD2*), Glutathione peroxidase 1 (*GPX1*), and Peroxiredoxin 3 (*PRDX3*) in our patient cohort**.

Many of the genetic disorders listed in Table 1 may present with PAI. Some of these conditions will have additional features and investigations will reveal abnormal findings, for example, adrenal cortex auto-antibodies or perturbed levels of 17-hydroxyprogesterone and very-long chain fatty acids to name a few [reviewed in Ref. (8)]. However, with recent discoveries of new genes and novel mutations in known genes causing PAI, it is becoming increasingly clear that considerable phenotypic overlap between genetic disorders occurs. This is applicable to the condition FGD, once thought to be fairly distinct from other causes of PAI (see Figure 1). FGD patients present with isolated glucocorticoid deficiency and normal mineralocorticoid production. Biochemical results in FGD point toward ACTH resistance, with serum cortisol often undetectable and extremely high plasma ACTH (11). As we learn more about the genetic causes of FGD, the boundaries between different PAI disorders or diagnostic features of FGD are being challenged (12–20).

Similar to many other endocrine disorders, obtaining an accurate diagnosis and understanding of the underlying pathology is essential for the management and treatment of the patient. Obtaining a genetic diagnosis will also enable prenatal screening, which in the case of CAH may inform or remove the need for antenatal treatment (21). Furthermore, a genetic diagnosis may directly alter therapeutic management of the patient removing the need for unnecessary medications lifelong (12).

In children, CAH is the commonest cause of PAI, causing 71.8% of cases in one report (22). In the cohort studied by Perry et al., this was followed by autoimmune causes (12.7%), adrenoleukodystrophy (3.9%), syndromic causes (4.9%), X-linked AHC (1%), and several were unexplained (5.8%). In this study, five international groups were involved in the mutational analysis of the various genes implicated in PAI (22).

Exome sequencing provides a robust technique by which coding variants can be identified and matched with their associated disease and complex phenotype (23). Exome sequencing was first used to identify the rare genetic mutation implicated in Miller Syndrome (23) and has been successfully used where linkage analysis has proven inadequate due to the rarity of the disease being investigated, the sporadic nature of the disease being investigated, or uninformative pedigrees. In addition, the MLL2 mutation was identified using exome sequencing as a cause of Kabuki syndrome, a rare genetic disorder. The causative genetic variant leading to Kabuki syndrome had proved difficult to determine due to the rarity of the disease, there only being approximately 400 cases worldwide (24). Further success in disease gene identification through WES includes the identification of the missense variant in SLC26A3 which results in congenital chloride diarrhea (25). In addition to using exome sequencing to identify a link between variants and disease, it has potential to be used as a diagnostic tool. In the case of congenital chloride diarrhea, exome sequencing identified the SLC26A3 variant, and future diagnosis of this condition could be achieved through exome sequencing (25). Even in cases demonstrating identification of disease causing variants using whole-genome sequencing, as in the case of hypercholesterolemia, these could often have been identified by exome sequencing (26). Many more examples, illustrating the emerging importance of exome sequencing, are summarized by Gilissen et al. (27). In PAI, similar to many other disorders, WES is often cheaper than the sequencing of all known candidate genes. Furthermore, given the lower cost associated with exome sequencing relative to whole-genome sequencing, WES sequencing may provide a robust and cost-effective screening and diagnostic tool for rare genetic conditions.

We have a cohort of >300 patients with FGD, 60% of them have a genetic diagnosis. We have previously used targeted and WES to identify novel causes of FGD in this collection (17, 18, 28). WES of a subset of our unsolved cases allowed for the screening of variants known to cause FGD as well as the possibility of discovering novel causative genes for PAI. Using PAI as an example, this paper offers a valuable commentary on the use of WES as a diagnostic tool in cases where the number of causative genes makes targeted gene sequencing more expensive than WES.

## Subject and Methods

### Sequencing of candidate genes in familial glucocorticoid deficiency

Patient genomic DNA was extracted from blood leukocytes. PCR was used to amplify regions of candidate genes implicated in FGD. All patients were screened for mutations in Melanocortin-2-receptor (*MC2R*), Melanocortin-2-receptor accessory protein (*MRAP*), and steroidogenic acute regulatory protein (*STAR*), the most commonly mutated genes in FGD, accounting for 50% of cases (Figure 1). Most patients underwent additional screening for mutations in nicotinamide nucleotide transhydrogenase (*NNT*), a recently discovered FGD causal gene (primer sequences available on request). After initial denaturation of template DNA at 95^o^C for 5 min, a touchdown thermal cycling program was used; 10 cycles of 95^o^C for 30 s, 65^o^C for the first cycle, this was then decreased by 1^o^C for each subsequent cycle for 10 cycles, and then 72^o^C for 1 min. This was then followed by 25 cycles of 95^o^C for 30 s, 55^o^C for 30 s and 72^o^C for 1 min. After the last cycle, there is an extension step at 72^o^C for 5 min. PCR products were sequenced using ABI Prism Big Dye Sequencing kit and an ABI 377 automated DNA sequencer (Applied Biosystems) as described in the manufacturer’s instructions.

Informed consent was obtained from affected individuals and/or their parents. Ethical approval for the study was obtained from the Outer North East London Research Ethics Committee, reference number 09/H0701/12.

### Exome sequencing

Whole-exome sequencing (WES) was performed on 43 patients with a diagnosis of FGD who were negative on screening for candidate genes (described above). This included many ‘cold cases’ referred to us as isolated glucocorticoid deficiency. In addition, one sibling (of patient 9) was sequenced for the causative gene and family members underwent Sanger sequencing of candidate variants to determine segregation.

**Figure 2:**
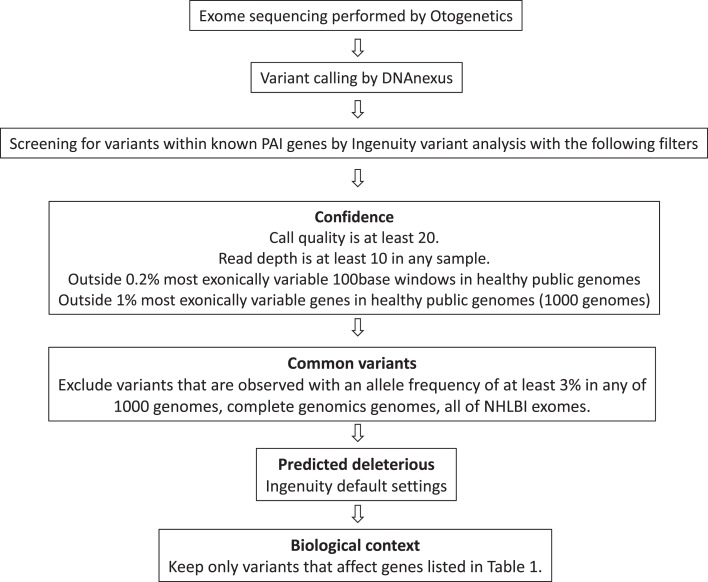
**Filtration strategy to screen variants from WES data**.

Exome sequencing was performed using the Agilent SureSelect all exon V4 capture and paired-end (2 × 100) sequencing on an Illumina HiSeq 2000 at Otogenetics (Norcross, GA). *First analysis pipeline*: initially sequencing read alignment, variant calling, and annotation were performed by DNAnexus (DNAnexus Inc.^1^, Mountain View, CA, USA), their Nucleotide-Level Variation analysis outputs were then screened with our list of genes via the DNAnexus Classic platform which permitted variants table viewing and filtering functionality. This platform no longer exists and more recently these data were reanalyzed uploading the vcf files to Ingenuity variant analysis^2^. *Second analysis pipeline:* the raw data were also reanalyzed, aligning to the H. Sapiens GRCh37–b37 (1000genomes Phase 1) reference genome with BWA-MEM FastQ Readmapper VCF files, generated by Vendor Human Exome GATK-Lite Variant Caller (Unified Genotyper) and uploaded to Ingenuity variant analysis. Single Nucleotide Polymorphisms, with threshold coverage of at least 10 reads on the respective nucleotide, were included in the analysis. The variant files from both analyses were screened for causal variants using Ingenuity variant analysis with the filtering strategy outlined in Figure 2. Sequence changes in PAI causal genes were confirmed by PCR designed to cover the affected region followed by Sanger sequencing (primer sequences available on request).

## Results

Mean target coverage across the exomes varied from 52.34 – 62.52X and > 90% of target bases were covered 10X. For both analysis pipelines, similar numbers of variants were called from 43 exomes combined, after confidence filtration the first analysis pipeline produced a total of 393,015 variants in 20,327 genes with many variants being common between samples whereas the second had 393,262 variants in 20,249 genes. 400 and 358 variants within our 20 PAI genes (Table 1) were called by first and second pipelines, respectively, and after further filtration (Figure 2) the following results were obtained. To be as inclusive as possible, data from the first analysis pipeline are detailed below except where stated (Tables 2 and 3).

**Table 2:** **Non-synonymous variants detected after Ingenuity filtration screening of the 20 PAI causal genes listed in Table 1**.

Patient number	Chromosome	Position (genome assembly: GRCh37.p13)	Reference allele	Sample allele	Variation type	Gene region	Gene symbol	Protein variant	Translation impact	SIFT function prediction	dbSNP ID/HGMD reference (where annotated)	NHLBI ESP frequency (%)	Zygosity	Causality
1	X	30326991	C	A	SNV	Exonic	NR0B1	E164*	Stop gain			0	Hemi	Causal
2	X	30326498		G	Insertion	Exonic	NR0B1	G329fs*60	Frameshift			0	Hemi	Causal
3	X	30326727	G	A	SNV	Exonic	NR0B1	Q252*	Stop gain			0	Hemi	Causal
4	X	30326343		A	Insertion	Exonic	NR0B1	Y380fs*9	Frameshift			0	Hemi	Causal
5	15	74631631	G	T	SNV	Exonic	CYP11A1	Q395K	Missense	Damaging		0	Het	Causal in comp het
5	15	74640307	G	A	SNV	Exonic	CYP11A1	R120Q	Missense	Damaging		0		Causal in comp het
6	15	74635318	C	T	SNV	Exonic	CYP11A1	T330T	Synonymous	Splicing		0	Het	Causal in comp het
6	15	74635368	C	T	SNV	Exonic	CYP11A1	E314K	Missense	Tolerated	rs6161	0.28	Het	Causal in comp het
7	15	74630315	A	T	SNV	Exonic	CYP11A1	*122Rext*68	Stop loss	Damaging		0		Causal in comp het
7	15	74635473	T		Deletion	Exonic	CYP11A1	I279Yfs*9	Frameshift	Damaging	CD050132	0		Causal in comp het
8	15	74630315	A	T	SNV	Exonic	CYP11A1	*122Rext*68	Stop loss	Damaging		0		Causal in comp het
8	15	74635473	T		Deletion	Exonic	CYP11A1	I279Yfs*9	Frameshift	Damaging	CD050132	0		Causal in comp het
9	15	74632009	G	A	SNV	Exonic	CYP11A1	A359V	Missense	Damaging	rs121912812	0	Homo	Causal homozygous
10	5	43613069	C	T	SNV	Exonic	NNT	R71*	Stop gain	Damaging		0	Het	Causal in comp het with pseudoexon event not detected by WES
11	5	43613069	C	T	SNV	Exonic	NNT	R71*	Stop gain	Damaging		0	Het	Causal in comp het with pseudoexon event not detected by WES
12	8	143956672		CT	Insertion	Exonic	CYP11B1	N394fs*37	Frameshift	Damaging	CI920932	0	Homo	Causal
13	21	45708296	C	T	SNV	Exonic	AIRE	R203*	Stop gain	Damaging	CM980043	0		Causal
14	21	45708296	C	T	SNV	Exonic	AIRE	R203*	Stop gain	Damaging	CM980043	0		Causal
15	12	53708169	C	T	SNV	Exonic	AAAS	W201	Stop gain	Damaging		0	Homo	Causal
16	12	53701478		A	Insertion	Exonic	AAAS	A480fs*16	Frameshift	Damaging		0	Homo	Causal
17	18	13885094	C	A	SNV	Exonic	MC2R	V142L	Missense	Damaging		0	Homo	Causal homozygous
18 to 30	No non-synonymous variants found in PAI genes													
31	6	32006924	C	T	SNV	Exonic	CYP21A2	L86F	Missense	Tolerated		0	Het	? Causal appears het because of pseudogene
32	6	32007344	T	A	SNV	Exonic	CYP21A2	Y161N	Missense	Damaging		0	Het	? Causal appears het because of pseudogene
33	6	32008500	G	A	SNV	Exonic	CYP21A2	A392T	Missense	Tolerated	rs202242769/CM071683	0	Het	? Causal appears het because of pseudogene
34	No non-synonymous variants found in PAI genes													
35	No non-synonymous variants found in PAI genes													
36	8	48874189	C	T	SNV	Exonic	MCM4	P62S	Missense	Tolerated		0	Het	? Significance on its own
37	18	13885438	G	C	SNV	Exonic	MC2R	P27R	Missense	Damaging	rs28926178	0.38	Het	? Significance on its own
38	8	38003911	G	A	SNV	Exonic	STAR	R121W	Missense	Damaging	rs34908868	0.12	Het	? Significance on its own
38	15	74635368	C	T	SNV	Exonic	CYP11A1	E314K	Missense	Tolerated	rs6161	0.28	Het	? Significance on its own
39	21	33671389	G	C	SNV	Splice Site; Intronic	MRAP	?	? No translation	Damaging	CS050119	0	Het	? Significance on its own
40	22	19864750	C	A	SNV	Exonic	TXNRD2	A485S	Missense	Damaging		0	Het	? Significance on its own
41	12	53714474	C	G	SNV	Exonic	AAAS	W42C	Missense	Tolerated		0	Het	? Significance on its own
41	21	33679047	T	C	SNV	Exonic	MRAP	M68T	Missense	Activating		0	Het	? Significance on its own
42	8	38003911	G	A	SNV	Exonic	STAR	R121W	Missense	Damaging	rs34908868	0.12	Het	? Significance on its own
42	8	48874175	T	A	SNV	Exonic	MCM4	V57E	Missense	Activating		0	Het	? Significance on its own
43	5	43616091	C	A	SNV	Exonic	NNT	L175M	Missense	Tolerated	rs145205428	0.08	Het	? Significance on its own

Key: HGMD, Human Gene Mutation Database; EVS, Exome Variant Server, NHLBI Exome Sequencing Project (ESP), Seattle, WA; SNV, single nucleotide variant; PAI, primary adrenal insufficiency; *, stop gain; ?, query.

**Table 3:** **Variants, including synonymous changes, detected after Ingenuity filtration screening of the 20 PAI causal genes listed in Table 1**.

Patient number	Chromo-some	Position (genome assembly: GRCh37.p13)	Reference allele	Sample allele	Variation type	Gene region	Gene symbol	Protein variant	Translation impact	SIFT function prediction	dbSNP ID/HGMD reference (where annotated)	NHLBI ESP frequency (%)	Zygosity	Causality	Variant detected with second pipeline analysis
1	8	48878772	C	T	SNV	Exonic	MCM4	S286S	Synonymous		rs17334388	0.4	Het	Not causal het and synonymous	Yes
1	8	48882392	A	G	SNV	Exonic	MCM4	P403P	Synonymous		rs17287656	0.38	Het	Not causal het and synonymous	Yes
**1**	**X**	**30326991**	**C**	**A**	**SNV**	**Exonic**	**NR0B1**	E164*	**Stop gain**			**0**	**Hemi**	**Causal**	**Yes**
**2**	**X**	**30326498**		**G**	**Insertion**	**Exonic**	**NR0B1**	G329fs*60	**Frameshift**			**0**	**Hemi**	**Causal**	**Yes**
3	8	48878849	C	T	SNV	Exonic	MCM4	T312M	Missense	Tolerated		0	Het	Not causal het	Yes
**3**	**X**	**30326727**	**G**	**A**	**SNV**	**Exonic**	**NR0B1**	Q252*	**Stop gain**			**0**	**Hemi**	**Causal**	**Yes**
4	8	143958492	C	G	SNV	Exonic	CYP11B1	R181P	Missense	Damaging	rs146105017	0.05	Het	? Significance	Yes
**4**	**X**	**30326343**		**A**	**Insertion**	**Exonic**	**NR0B1**	Y380fs*9	**Frameshift**			**0**	**Hemi**	**Causal**	**Yes**
5	8	38006217	C	T	SNV	Exonic	STAR	L40L	Synonymous		rs138786388	0.29	Het	Not causal het and synonymous	Yes
**5**	**15**	**74631631**	**G**	**T**	**SNV**	**Exonic**	**CYP11A1**	**Q395K**	**Missense**	**Damaging**		**0**	**Het**	**Causal in comp het**	**Yes**
**5**	**15**	**74640307**	**G**	**A**	**SNV**	**Exonic**	**CYP11A1**	**R120Q**	**Missense**	**Damaging**		**0**		**Causal in comp het**	**Yes**
**6**	**15**	**74635318**	**C**	**T**	**SNV**	**Exonic**	**CYP11A1**	**T330T**	**Synonymous**	**Splicing**		**0**	**Het**	**Causal in comp het**	**Yes**
**6**	**15**	**74635368**	**C**	**T**	**SNV**	**Exonic**	**CYP11A1**	**E314K**	**Missense**	**Tolerated**	**rs6161**	**0.28**	**Het**	**Causal in comp het**	**Yes**
**7**	**15**	**74630315**	**A**	**T**	**SNV**	**Exonic**	**CYP11A1**	*122Rext*68	**Stop loss**	**Damaging**		**0**		**Causal in comp het**	**Yes**
**7**	**15**	**74635473**	**T**		**Deletion**	**Exonic**	**CYP11A1**	I279Yfs*9	**Frameshift**	**Damaging**	**CD050132**	**0**		**Causal in comp het**	**Yes**
**8**	**15**	**74630315**	**A**	**T**	**SNV**	**Exonic**	**CYP11A1**	*122Rext*68	**Stop loss**	**Damaging**		**0**		**Causal in comp het**	**Yes**
**8**	**15**	**74635473**	**T**		**Deletion**	**Exonic**	**CYP11A1**	I279Yfs*9	**Frameshift**	**Damaging**	**CD050132**	**0**		**Causal in comp het**	**Yes**
9	12	53708092	A	G	SNV	Exonic	AAAS	L227L	Synonymous		rs80027466	0.49	Het	Not causal het and synonymous	Yes
**9**	**15**	**74632009**	**G**	**A**	**SNV**	**Exonic**	**CYP11A1**	**A359V**	**Missense**	**Damaging**	**rs121912812**	**0**	**Homo**	**Causal homozygous**	**Yes**
**10**	**5**	**43613069**	**C**	**T**	**SNV**	**Exonic**	**NNT**	R71*	**Stop gain**	**Damaging**		**0**	**Het**	**Causal in comp het with pseudoexon event not detected by WES**	**Yes**
10	11	2905982	A	T	SNV	Exonic	CDKN1C	I235I	Synonymous			0	Het	Not causal het and synonymous	Yes
**11**	**5**	**43613069**	**C**	**T**	**SNV**	**Exonic**	**NNT**	R71*	**Stop gain**	**Damaging**		**0**	**Het**	**Causal in comp het with pseudoexon event not detected by WES**	**Yes**
11	5	43613188	T	G	SNV	Exonic	NNT	G110G	Synonymous		rs200396139	0	Het	Not causal het and synonymous	No
12	5	43613234	G	A	SNV	Exonic	NNT	V126I	Missense	Damaging		0	Het	? Significance	Yes
12	5	43700321	A	G	SNV	Exonic	NNT	I993V	Missense	Damaging	rs78818665	0.43	Het	? Significance	Yes
**12**	**8**	**143956672**		**CT**	**Insertion**	**Exonic**	**CYP11B1**	N394fs*37	**Frameshift**	**Damaging**	**CI920932**	**0**	**Homo**	**Causal**	**Yes**
**13**	**21**	**45708296**	**C**	**T**	**SNV**	**Exonic**	**AIRE**	R203*	**Stop gain**	**Damaging**	**CM980043**	**0**		**Causal**	**Yes**
**14**	**21**	**45708296**	**C**	**T**	**SNV**	**Exonic**	**AIRE**	R203*	**Stop gain**	**Damaging**	**CM980043**	**0**		**Causal**	**Yes**
15	8	48878772	C	T	SNV	Exonic	MCM4	S286S	Synonymous		rs17334388	0.4	Het	Not causal het and synonymous	Yes
15	8	48882392	A	G	SNV	Exonic	MCM4	P403P	Synonymous		rs17287656	0.38	Het	Not causal het and synonymous	Yes
15	8	48883160	G	A	SNV	Exonic	MCM4	L508L	Synonymous			0	Het	Not causal het and synonymous	Yes
15	8	48885434	TC	GA	Substitution	Exonic	MCM4	L649R	In-frame	Damaging		0	Het	Not causal het	Yes
**15**	**12**	**53708169**	**C**	**T**	**SNV**	**Exonic**	**AAAS**	**W201**	**Stop gain**	**Damaging**		**0**	**Homo**	**Causal**	**Yes**
15	15	74631994	T	C	SNV	Exonic	CYP11A1	Q364R	Missense	Tolerated	rs57982762	0.02	Het	? Significance	Yes
**16**	**12**	**53701478**		**A**	**Insertion**	**Exonic**	**AAAS**	A480fs*16	**Frameshift**	**Damaging**		**0**	**Homo**	**Causal**	**Yes**
**17**	**18**	**13885094**	**C**	**A**	**SNV**	**Exonic**	**MC2R**	**V142L**	**Missense**	**Damaging**		**0**	**Homo**	**Causal homozygous**	**Yes**
17	22	19868170	G	A	SNV	Exonic	TXNRD2	S386F	Missense	Damaging		0	Het	? Significance	Yes
18 to 24															
25	5	43613188	T	G	SNV	Exonic	NNT	G110G	Synonymous		rs200396139	0	Het	Not causal het and synonymous	No
26	5	43613188	T	G	SNV	Exonic	NNT	G110G	Synonymous		rs200396139	0	Het	Not causal het and synonymous	No
27	5	43613188	T	G	SNV	Exonic	NNT	G110G	Synonymous		rs200396139	0	Het	Not causal het and synonymous	No
27	9	127262606	G	A	SNV	Exonic	NR5A1	Y211Y	Synonymous		rs374363746	0.13	Het	Not causal het and synonymous	Yes
28	6	32006337	C	A	SNV	Exonic	CYP21A2	P46P	Synonymous		rs6464	0	Het	Not causal het and synonymous	Yes
29	8	143960555	G	A	SNV	Exonic	CYP11B1	D96D	Synonymous		rs5284	0.04	Het	Not causal het and synonymous	Yes
30	18	13885083	G	A	SNV	Exonic	MC2R	R145R	Synonymous		rs369830440	0.02	Het	Not causal het and synonymous	Yes
**31**	**6**	**32006924**	**C**	**T**	**SNV**	**Exonic**	**CYP21A2**	**L86F**	**Missense**	**Tolerated**		**0**	**Het**	**? Causal appears het because of pseudogene**	**Yes**
32	5	43613188	T	G	SNV	Exonic	NNT	G110G	Synonymous		rs200396139	0	Het	Not causal het and synonymous	No
**32**	**6**	**32007344**	**T**	**A**	**SNV**	**Exonic**	**CYP21A2**	**Y161N**	**Missense**	**Damaging**		**0**	**Het**	**? Causal appears het because of pseudogene**	**Yes**
32	22	19868177	C	T	SNV	Exonic	TXNRD2	G384S	Missense	Tolerated	rs192869629	0.41	Het	? Causal? comp het	Yes
33	5	43613188	T	G	SNV	Exonic	NNT	G110G	Synonymous		rs200396139	0	Het	Not causal het and synonymous	No
**33**	**6**	**32008500**	**G**	**A**	**SNV**	**Exonic**	**CYP21A2**	**A392T**	**Missense**	**Tolerated**	**rs202242769/CM071683**	**0**	**Het**	**? Causal appears het because of pseudogene**	**Yes**
34	11	2906607	TC	AT	Substitution	Exonic	CDKN1C	D38I	In-frame			0	Het	Sequencing anomaly	no
35	11	2905964	GG	TT	Substitution	Exonic	CDKN1C	A241E	In-frame			0	Het	Sequencing anomaly	No
35	11	2905969	CGGG	ATCT	Substitution	Exonic	CDKN1C	P239_A240delinsRS	In-frame			0	Het	Sequencing anomaly	No
36	8	48874189	C	T	SNV	Exonic	MCM4	P62S	Missense	Tolerated		0	Het	? Significance on its own	Yes
36	11	2906501	GCCC	AGAT	Substitution	Exonic	CDKN1C	G62S	In-frame	Tolerated		0	Het	Sequencing anomaly	No
36	11	2906509	GCG	AGA	Substitution	Exonic	CDKN1C	P70L	In-frame	Damaging		0	Het	Sequencing anomaly	No
37	9	127245072	GG	TC	Substitution	Exonic	NR5A1	N450_L451delinsKM	In-frame			0	Het	Sequencing anomaly	No
37	18	13885438	G	C	SNV	Exonic	MC2R	P27R	Missense	Damaging	rs28926178	0.38	Het	? Significance on its own	Yes
38	5	43616018	G	T	SNV	Exonic	NNT	T150T	Synonymous			0	Het	Not causal het and synonymous	Yes
38	8	38003911	G	A	SNV	Exonic	STAR	R121W	Missense	Damaging	rs34908868	0.12	Het	? Significance on its own	Yes
38	15	74635368	C	T	SNV	Exonic	CYP11A1	E314K	Missense	Tolerated	rs6161	0.28	Het	? Significance on its own	Yes
39	21	33671389	G	C	SNV	Splice Site; Intronic	MRAP	?	? No translation	Damaging	CS050119	0	Het	? Significance on its own	Yes
40	22	19864750	C	A	SNV	Exonic	TXNRD2	A485S	Missense	Damaging		0	Het	? Significance on its own	Yes
41	12	53714474	C	G	SNV	Exonic	AAAS	W42C	Missense	Tolerated		0	Het	? Significance on its own	Yes
41	21	33679047	T	C	SNV	Exonic	MRAP	M68T	Missense	Activating		0	Het	? Significance on its own	Yes
42	8	38003911	G	A	SNV	Exonic	STAR	R121W	Missense	Damaging	rs34908868	0.12	Het	? Significance on its own	Yes
42	8	48874170	T	G	SNV	Exonic	MCM4	P55P	Synonymous			0	Het	? Significance on its own	No
42	8	48874175	T	A	SNV	Exonic	MCM4	V57E	Missense	Activating		0	Het	? Significance on its own	No
43	5	43616091	C	A	SNV	Exonic	NNT	L175M	Missense	Tolerated	rs145205428	0.08	Het	? Significance on its own	No

*Key: HGMD, Human Gene Mutation Database; EVS, Exome Variant Server, NHLBI Exome Sequencing Project (ESP), Seattle, WA; SNV, single nucleotide variant; PAI, primary adrenal insufficiency*.

Variants highlighted in bold are believed to be causal; *, stop gain; ?, query.

### Positive genetic diagnosis

After Ingenuity filtration screening (Figure 2) of the 20 PAI causal genes listed in Table 1, 51 variants in 12 genes remained (Table 2), with no variants in *ABCD1, CDKN1C, CYP17A1, GPX1, HSD3B2, NR5A1, POR*, or *PRDX3* being detected. When rare synonymous variants were included, to encompass silent changes that altered splicing (as in patient 6 below), 69 variants were discovered (Table 3) with no variants in *ABCD1, CYP17A1, GPX1, HSD3B2, POR*, or *PRDX3* being detected.

We made a genetic diagnosis in 17 patients plus one affected sibling (of patient 9) who did not undergo WES, identifying mutations in the following genes: *NR0B1* (four patients), *CYP11A1* (five patients + one sib), *AAAS* (two patients), *MC2R* (one patient), *CYP11B1* (one patient), *AIRE* (two patients), and *NNT* (two siblings) (patients 1–17 in Table 2). Where possible, segregation of the variant(s) with the disease was confirmed by direct Sanger sequencing of the index case and family members.

Patients 1–4, all males, were hemizygous for novel mutations in *NR0B1*, these were stop gain or frameshift mutations and therefore likely to be causal. Patients 5–8 had compound heterozygous variants in *CYP11A1* that segregated with the disease; patient 5 was compound heterozygous for two novel missense mutations; and patient 6 was compound heterozygous for E314K (MAF 0.001%) and a silent T330T variant at the end of exon 5 of *CYP11A1* which we showed, by cDNA analysis, resulted in aberrant splicing. Siblings 7 and 8 were compound heterozygous for I279Yfs*9, previously reported in lipoid congenital adrenal hyperplasia [LCAH; Hiort et al. (29); (29)] and a novel, stop loss mutation *122Rext*68. Patient 9 and a sibling had a homozygous mutation, A359V in *CYP11A1* which has previously been seen in LCAH with XY sex reversal (30).

In siblings 10 and 11, we detected a heterozygous stop gain mutation, R71* in NNT, no other variants were detected by either WES or Sanger sequencing of the other *NNT* exons; however, the presence of a stop mutation on one allele in both siblings prompted further investigations including cDNA analysis which uncovered a pseudoexon inclusion event on the other allele. A pseudoexon is a potential exon within intronic regions of pre-mRNA that is not normally spliced into mature mRNA, it contains sequences similar to 5′ and 3′ splice-site consensus sequences, but is not normally recognized by the cellular splicing machinery. In this case the pseudoexon inclusion results in a frameshift and the introduction of a premature stop codon (31). Patient 12 had a homozygous frameshift mutation in *CYP11B1*, previously described in CAH (32), but also bore novel variants in *NNT* (see below). Siblings 13 and 14 were homozygous for a novel, stop gain mutation in *AIRE*, the gene responsible for APECED/APS1. Patients 15 and 16 had homozygous stop gain and frameshift mutations, respectively, in *AAAS*, causal for Triple A syndrome. Finally, patient 17 was homozygous for a previously described MC2R mutation (33), which had been missed on conventional sequencing. In summary, for patients 1–17 variants in PAI causal genes were detected which were likely to be responsible for their disease.

### Incidental mutations in solved cases

Several of the above patients, with a presumed genetic diagnosis, bore heterozygous changes in other PAI genes (Table 2); patient 3 carried p.T312M in *MCM4*, patient 4 carried p.R181P in *CYP11B1*, patient 12 had three variants, p.L361D in *CYP17A1*, and p.V126I and p.I993V in *NNT*, patient 15 had two, p.Q364R in *CYP11A1* and p.L649R in *MCM4*, and patient 17 had two, p.H24_Y25delinsQI in *NR5A1* and p.S386F in *TXNRD2*. The contribution of these variants to the burden of their disease is uncertain.

### No genetic diagnosis

By contrast, a genetic diagnosis was not confirmed in patients 18–43. For patients 18–24, no variants were detected in the genes screened; patients 25–30 only possessed heterozygous, synonymous variants in the 16 genes screened. Patients 31–33 had heterozygous changes in *CYP21A2* (or its pseudogene*/CYP21A1P*) consistent with gene conversion events but needing further investigations to confirm this. Patients 34–43 had heterozygous, missense or frameshift variants in genes known to cause PAI, but in each case only a single allele was affected. The frameshift variants found in *CDKN1C* and *NR5A1* in patients 34–37 proved to be sequencing anomalies and were excluded by the second analysis pipeline, whereas the missense variants in *MCM4*, *MC2R, NNT, STAR, CYP11A1, MRAP, TXNRD2*, and *AAAS* seen in patients 37–43 were confirmed by Sanger sequencing. The G110G and L175M *NNT* variants, the P55P and V57E *MCM4* variants, the N450_L451delinsKM in *NR5A1*, and all *CDKN1C* variants were missed by the second analysis (Table 3 last column). A low allele fraction for the variant nucleotide accounted for the error in calling variants found in *NNT* and *MCM4*, but for the changes in *NR5A1* and *CDKN1C* the sequence changes were not confirmed by Sanger sequencing and on close inspection in Integrative Genomics Viewer (IGV, Broad Institute^3^) were due to misalignment of sequencing reads within repetitive sequences. Sanger sequencing of all exons of the affected gene was carried out in each case for patients 37–43 to determine whether a causal variant could be discovered on the other allele but without success. The inheritance pattern for these genes is autosomal recessive and the causative mutations are loss of function, this would therefore suggest that either these are incidental mutations not contributing to disease, or there are regulatory region or intronic variants on the other allele in each of these cases, similar to the scenario in *NNT* for patients 10 and 11. This requires mRNA/cDNA analysis for which samples are unavailable at this time.

## Discussion

Exome sequencing has been used to successfully identify novel variants in diseases causing adrenal insufficiency. In the case of FGD, 50% of cases are attributable to mutations in one of three genes: *MC2R*, *MRAP*, and *STAR*, which were linked to the disorder by traditional linkage and sequencing methods, but with the advent of next generation sequencing technologies, three further genes have been discovered and more causative genes may exist (Figure 1) (14, 17, 18, 28). Targeted exome sequencing was used to identify the first variants in *NNT* and subsequently 21 variants were identified as causal in FGD (18). In addition, exome sequencing was also used to identify the c.71-1insG variant in the *MCM4* gene which results in the syndrome of adrenal failure, short stature, and natural killer (NK) cell deficiency in Irish Travelers (17) and the p.Y447* variant in *TXNRD2* responsible for FGD in a large Kashmiri kindred (28). WES provided a rapid genetic diagnosis in 17/43 sequenced ‘FGD’ patients, in most cases identifying an alternative genetic cause of their PAI (14/17). For the remaining 60%, the gene defect may be within intronic/regulatory regions not covered by WES or may be in gene(s) representing novel etiologies.

In the case of suspected PAI, there are many candidate genes implicated (Table 1). Exome sequencing offers a rapid, cheap, and convenient sequencing technique as the exons of a large repertoire of candidate genes can be screened in one sequencing run. The findings in this cohort highlight the complex nature of adrenal disease and the range of differential diagnoses that may arise. It is critical to determine the underlying genetic cause in adrenal insufficiency since this may change on-going treatment and management. Patients with partial loss-of-function mutations in *STAR* or *CYP11A1* presenting an adrenal-only phenotype may have associated fertility problems (14), such patients require close monitoring as they may have a window of opportunity for fertility that is lost over time. Early genetic counseling should be offered to families and discussion about options for preservation of fertility is required. Furthermore, the identification of *NROB1* mutations in some of our ‘FGD’ cohort would allow clinicians to recognize and manage future issues saving their patients the anguish of later unexplained infertility.

Furthermore, adopting exome sequencing as a standard screening tool would allow for the identification of variants which may have later onset as is the case with the *MCM4* variant leading to mild cortisol deficiency in childhood which declines with age after a period of normal adrenal function (17) or *TXNRD2* variants which may have an extremely variable age of onset (28). Such an approach also increases the probability of new gene causality being discovered with the accrual of patients without mutations in known PAI genes.

There are, however, limitations to exome sequencing as a screening tool for known disease gene variants. The exome is thought to represent approximately 1% of the human genome, thus leaving a large section of the DNA unchecked (34). Although the exome is thought to account for 85% of disease causing mutations (25) the true incidence is unknown because of the difficulty of sequencing and characterizing intronic variants. Our results, both the discovery of a pseudoexon inclusion event and patients with single heterozygous changes, suggest the possibility that this may occur more frequently than previously recognized (31). For patients 10 and 11, the finding of a stop gain mutation in *NNT* in both siblings led us to invest the time to investigate the other allele, this detected a pseudoexon inclusion event deep within intron 20 (1129 bp from exon 20), a region not covered by WES. This might have been missed if it had not been coupled with a known deleterious mutation on the other allele or if it had been homozygous. Similarly, compound heterozygous intronic mutations would be missed by WES. Whole-genome sequencing would disclose such cases but the consequence of such changes can be hard to predict. Most significantly, disease causing variants which lie in the non-coding promoter and intronic sequence such as is the case with apoE4 implicated in Alzheimer’s disease are also not detected by exome sequencing (35, 36). In addition, micro RNA genes which often silence expression of protein encoding genes are often found within introns. The percentage of disease causing mutations in exonic sequences may decrease with the application of whole-genome sequencing in patients and the improvement of tools for consequence prediction.

Even when dealing with an exonic variant the prediction of its pathogenicity is not trivial and this is exacerbated when the variant is outside coding sequences. Many computational methods have been developed to address this but concordance between the results generated by such programs is poor (37). Therefore, for optimal assessment of a variants effect more than one prediction program should be utilized and, where possible, functional studies should be undertaken. Similarly, multiple software tools for alignment and variant calling are available, and each can generate different results, here again it is recommended that more than one algorithm be employed when analyzing exome data (38, 39). Particularly low concordance rates have been reported for INDELs detected by different pipelines and sequencing platforms (40, 41) stressing the need to carry out multiple analyses on data sets. Depth of coverage is also a consideration, at a mean on-target read depth of 20X, commonly achieved in rare disease exome sequencing, it is predicted that 5–15% of heterozygous and 1–4% of homozygous single nucleotide variants (SNVs) in the targeted regions will be missed (42). Sulonen et al. showed that a minimum of 11X coverage was required to make a heterozygote genotype call with 99% accuracy, whereas homozygous calls can be accurate at much lower coverage (43).

Our exome data, ordered at a depth of 30X for economy, has many exons which are not covered 30X. For new gene discovery, this depth of coverage may be sufficient but it is clearly not ideal for diagnosis where a more appropriate depth might be 100X. One way to increase depth of coverage is to use a custom enrichment panel to go forward for next-generation sequencing. Many companies are now introducing clinical exome panels designed to capture the exons of disease causing genes, e.g., OneSeq and SureSelectXT Clinical Research Exome (Agilent) which may concomitantly detect genome-wide CNVs, copy-neutral LOH (cnLOH), SNPs, and indels in one target enrichment capture.

Additional disadvantages of WES include the fact that it cannot detect epigenetic changes, or mutations that do not alter the sequence, for instance, translocations, inversions, and repeats such as in Huntington’s disease. Consequently, exome sequencing cannot easily be used to assess gene copy number dependent disease susceptibility, as non-sequence alteration changes are not recognized. However, algorithms to address this have been and are being developed (44–46) and this is important as variations in copy number can affect the severity or susceptibility to a disease. Furthermore, silent changes, which may not affect amino acid sequence but alter splicing either directly or by alteration of splice enhancer/repressor sites, may be ignored as they are excluded from analysis by common filtration strategies. Difficulties also arise in WES in distinguishing between genes with extremely similar nucleotide sequences, where a gene possesses a paralog or pseudogene(s) with very similar sequence. For PAI, there are at least two such complications; to differentiate between *CYP21A2* and its pseudogene *CYP21A1P* and distinguishing between the paralogs *CYP11B1* and *CYP11B2*. This is usually achieved by PCR amplification strategies using primers spanning the distinguishing nucleotides, which are further apart than is convenient for standard PCR. Long-range PCR using allele-specific primers has been adopted by several groups to address this followed by sequencing of smaller sub-fragments (47). Currently such analyses will need to be applied in addition to WES. This report highlights the potential for exome sequencing to be used as a first pass diagnostic tool for conditions of adrenal insufficiency. However, there are ethical considerations and data protection issues which must be considered before using exome sequencing as a standard diagnostic tool (48). Exome sequencing for adrenal disease may lead to the identification of a variant implicated in a disease unrelated to that which the initial screening aimed to address. This leads to the question of whether the patient should be informed of variants which may have disease associations outside of the initial frame of consideration. It may seem appropriate that a patient should be notified if there is a strong monogenic link, but often diseases are polygenic, loosely associated, or linked only with susceptibility such as the case with the BRCA1 genes (49). Furthermore, not all diseases that have a characterized genetic cause have a treatment. It is important to consider whether it is appropriate or desirable for the patient to know about a disease for which there is no treatment. Any decision to notify of a genetic condition, whether treatment is available or not, would have to involve genetic counseling, and this would have to be an essential component of exome sequencing as a diagnostic tool. The American College of Medical Genetics (ACMG) guidelines suggest that laboratories performing diagnostic exome sequencing should report alterations in genes from among a provided minimum list of genes. This list includes 57 genes associated with 25 disorders which were chosen based on availability of preventative measures and treatment options (50, 51).

This highlights one of the abiding difficulties for WES diagnosis, if a novel variant is found in a disease causing gene how do we predict its consequence? In order to say it is causal for disease, we need to show the variant is loss- or gain-of-function which requires time-consuming and labor-intensive experiments, to check the (dys)function of the putative mutation. Such additional functional analyses will slow down the diagnostic process until such time as variant/protein prediction tools improve.

In summary, diagnostic WES provides rapid, cheap, discriminatory sequencing of all exonic sequences of known causal genes in one hit, thereby removing the necessity for a single, candidate gene, Sanger sequencing approach. However, WES does not cover intronic sequences which may harbor deleterious mutations, it does not allow easy detection of CNV, transversions, translocations, gene conversions (such as that frequently seen in *CYP21A2* in CAH), or gene fusions (seen between *CYP11B1/B2*) and it cannot predict the functional consequence of novel variant(s). In our hands WES provided a rapid, putative genetic diagnosis in 40% of our patients, in most cases the defect was not in a known FGD gene thereby highlighting both the difficulty of phenotypically distinguishing between disorders of PAI and the utility of WES as a tool to achieve this.

Take home message: Despite the limitations, exome sequencing still provides a powerful tool in the diagnosis of single gene and increasingly multigene disorders (25, 27). This report highlights the potential for WES to be a diagnostic aid in the clinical setting with the understanding that it cannot currently replace other genetic testing. In the case of PAI, and probably many other conditions, it would prove a useful first pass screen for rapid genetic diagnosis. Combined with copy number analysis, autoimmunity screening and specific screening where pseudogenes/paralogs exist, WES provides a useful tool in the diagnostic arsenal for endocrine clinicians. It is likely that WES will be adopted for the diagnosis of other endocrine disorders, such as growth hormone insensitivity and Kallmann’s syndrome, whereby the number of genes and/or the oligogenic nature of the inheritance pattern make WES a cheaper alternative than single gene sequencing.

## Conflict of Interest Statement

The authors declare that the research was conducted in the absence of any commercial or financial relationships that could be construed as a potential conflict of interest.

## Footnotes

^1^www.dnanexus.com

^2^www.ingenuity.com

^3^https://www.broadinstitute.org/igv/

## Web Resources

dbSNP, www.ncbi.nlm.nih.gov/SNP/

DNAnexus, https://dnanexus.com/

Exome Variant Server, NHLBI Exome Sequencing Project (ESP), Seattle, WA, http://evs.gs.washington.edu/EVS/[accessed November 2014].

Online Mendelian Inheritance in Man (OMIM), http://www.omim.org/

The Human Gene Mutation Database: http://www.hgmd.cf.ac.uk/ac/index.php

Ingenuity variant analysis: http://www.ingenuity.com/

Otogenetics: http://www.otogenetics.com/

GATK-Lite Variant Caller (Unified Genotyper): https://www.broadinstitute.org/gatk/gatkdocs/org_broadinstitute_gatk_tools_walkers_genotyper_UnifiedGenotyper.php

IGV: https://www.broadinstitute.org/igv/

## Abbreviations

AHC, adrenal hypoplasia congenital; AI, adrenal insufficiency; APECED/APS1, autoimmune polyendocrinopathy-candidiasis-ectodermal dystrophy/autoimmune polyendocrinopathy syndrome type I; CAH, congenital adrenal hypoplasia; FGD, familial glucocorticoid deficiency; HGMD, The Human Gene Mutation Database; IMAGE, IUGR, metaphyseal dysplasia, genital anomalies; IUGR, intrauterine growth retardation; IVA, Ingenuity variant analysis; LCAH, lipoid congenital adrenal hypoplasia; MAF, minor allele frequency; N/A, not applicable; NK, natural killer; OMIM, online Mendelian inheritance in man; PAI, primary adrenal insufficiency; X-ALD, X-linked adrenoleukodystrophy.
